# Room for Improvement in Conducting and Reporting Non-Inferiority Randomized Controlled Trials on Drugs: A Systematic Review

**DOI:** 10.1371/journal.pone.0013550

**Published:** 2010-10-27

**Authors:** Grace Wangge, Olaf H. Klungel, Kit C. B. Roes, Anthonius de Boer, Arno W. Hoes, Mirjam J. Knol

**Affiliations:** 1 Division of Pharmacoepidemiology and Clinical Pharmacology, Utrecht Institute for Pharmaceutical Sciences (UIPS), Utrecht University, Utrecht, The Netherlands; 2 University Medical Center Utrecht, Julius Center for Health Sciences and Primary Care, Utrecht, The Netherlands; Universidad Peruana Cayetano Heredia, Peru

## Abstract

**Background:**

A non-inferiority (NI) trial is intended to show that the effect of a new treatment is not worse than the comparator. We conducted a review to identify how NI trials were conducted and reported, and whether the standard requirements from the guidelines were followed.

**Methodology and Principal Findings:**

From 300 randomly selected articles on NI trials registered in PubMed at 5 February 2009, we included 227 NI articles that referred to 232 trials. We excluded studies on bioequivalence, trials on healthy volunteers, non-drug trials, and articles of which the full-text version could not be retrieved. A large proportion of trials (34.0%) did not use blinding. The NI margin was reported in 97.8% of the trials, but only 45.7% of the trials reported the method to determine the margin. Most of the trials used either intention to treat (ITT) (34.9%) or per-protocol (PP) analysis (19.4%), while 41.8% of the trials used both methods. Less than 10% of the trials included a placebo arm to confirm the efficacy of the new drug and active comparator against placebo, and less than 5.0% were reporting the similarity of the current trial with the previous comparator's trials. In general, no difference was seen in the quality of reporting before and after the release of the CONSORT statement extension 2006 or between the high-impact and low-impact journals.

**Conclusion:**

The conduct and reporting of NI trials can be improved, particularly in terms of maximizing the use of blinding, the use of both ITT and PP analysis, reporting the similarity with the previous comparator's trials to guarantee a valid constancy assumption, and most importantly reporting the method to determine the NI margin.

## Introduction

In the drug development process, the randomized controlled trial (RCT) can have a superiority, equivalence or a non-inferiority design. A superiority trial aims to demonstrate the superiority of a new therapy compared to an active comparator or a placebo, while an equivalence trial aims to demonstrate that a new therapy is equivalent (within margins) to its active comparator. In non-inferiority (NI) trials, the aim is to show that the new treatment is not worse than the comparator, which typically is an active drug.

NI trials can be used in a situation when a new drug considered has a similar efficacy profile as its comparator but may offer other advantages over the existing drug such as a novel method of administration or a better safety profile. In a regulatory setting, NI trials can be used to provide primary, but indirect, evidence of efficacy of a novel drug in cases where a placebo control treatment is not ethically justified.[1], [2]

Critics have pointed at various drawbacks of NI trials, questioning whether they are really useful. Some argue that NI trials only benefit pharmaceutical industry as they allow drugs without additional clinical efficacy to enter the market.[3], [4] However, as argued by Jones et.al, in some cases the new treatment may have no direct advantage but may present an alternative or second line therapy.[5]

From a methodological perspective, compared to superiority trials, NI trials have methodological issues in design and analysis that can influence proper inference. First, the value of blinding in NI trial is under debate, especially if the endpoints are subjective.[6] In a superiority trial, a blinded investigator who has a preliminary belief in superiority of the test drug cannot manipulate the results to support his belief. On the contrary, in an NI trial, the blinded investigator with a preliminary belief in non-inferiority of the test drug can bias the result by assigning similar ratings to the treatment responses of all patients. Others argued that blinding is still important to show the differences between drugs in NI trials.[7] Second, there are different methods to determine the NI margin and there are debates on whether the margin should be determined based on statistical or clinical considerations. Third, although there is a degree of consensus that non-inferiority should be shown for both the intention-to-treat (ITT) and per-protocol (PP) analysis sets, it is not clear whether this will be conservative or anti-conservative in a particular situation.[6], [7] Fourth, a difficulty in interpreting NI trials is their lack of ability to distinguish an effective drug from an ineffective drug i.e. assay sensitivity [7], [8], without relying on evidence outside the trial. A drug is considered effective if it shows a significant treatment effect compared with placebo. An additional placebo arm is recommended to confirm assay sensitivity [2], [6], [9]. However, this is often impossible due to ethical reasons. Last, the validity of the historical data that was used as the reference for the current trial, i.e. constancy assumption, is a critical point in the interpretation of NI trials. Related to the last issue, the CONSORT statement has recommended authors to mention whether the eligibility criteria, interventions and outcomes are identical or very similar to any trial that established efficacy of the reference treatment.[10] The effort is encouraged to support the validity of the constancy assumption.

The ICH E9 [11], the ICH E10 [8], CHMP guidelines [12] and the extension of the CONSORT statement on NI trials[10] are the currently available guidelines for the appropriate conduct and report of NI trials. We summarized the guidelines' recommendations on the five methodological issues described above in Table 1. Furthermore, we included the FDA draft guideline on NI trials [13] in Table 1 for consideration. The draft FDA guideline is not in effect yet and still open for changes (as per 18^th^ March 2010).

**Table 1:** The requirements in the guidelines for conducting and reporting NI trials.

Issues in NI trials	Requirements in the guidelines
***Blinding method***	Blinding is necessary to minimize bias *(ICH E9 and E10)*It is critical to provide reassurance and procedures that ensure maintenance of blinding *(draft FDA guideline on NI trial 2010)*
***NI margin***	An acceptable non-inferiority margin should be defined *(ICH E10, CPMP/EMEA 2000)* Should be pre-specified, and can be no larger than the presumed entire effect of the active control in the NI trial *(draft FDA guideline on NI trial 2010)* Should be specified in publication *(CONSORT statement extension, 2006)*
***Method to determine NI margin***	The determination of the margin in a non-inferiority trial is based on both statistical reasoning and clinical judgment *(ICH E10)* Margin is chosen by defining the largest difference that is clinically acceptable, so that a difference bigger than this would matter in practice *(CPMP/EMEA 2000)* The NI margin should be generally identified based on previous experience in placebo-controlled trials of adequate design under conditions similar to those planned for the current trial, but could also be supported by dose response or active control superiority studies.*(ICH E10, CHMP/EMEA 2005)* Fixed margin method (two CIs method) is recommended. It is referred to as fixed because the past studies comparing the drug with placebo are used to derive a single fixed value for statistical margin, even though this value is based on results of placebo-controlled trials (one or multiple trials versus placebo) that have a point estimate and confidence interval for the comparison with placebo. This approach is relatively conservative, as it keeps separate the variability of estimates of the treatment effect in the historical studies and the variability observed in the NI trial, and uses a fixed value for the estimate of the control effect based on historical data (the 90% or 95% CI lower bound), a relatively conservative estimate of the control drug effect. *(draft FDA guideline on NI trial 2010)* should be specified in publication *(CONSORT statement extension, 2006)*
***Type of statistical analysis***	Use of the full analysis set is generally not conservative and its role should be considered very careful *(ICH E9)* Both ITT and PP have equal importance *(CPMP/EMEA 2000)* Important to conduct both ITT and as-treated analyses. Differences in results using the two analyses will need close examination. (*draft FDA guideline on NI trial 2010*)
***Assay sensitivity***	A trial should have the ability to distinguish an effective from an ineffective drug *(ICH 10, draft FDA guideline on NI trial 2010)* A three-armed trial with test, reference and placebo allows some within-trial validation of the choice of non-inferiority margin and should be used wherever possible.(*CHMP/EMEA 2005, draft FDA guideline on NI trial 2010*)
***Constancy assumption***	The similarity of the new trial to the historical trial should be sufficient (*CHMP/EMEA 2005, draft FDA guideline on NI trial 2010*)
***Similarity with trial of reference treatment***	The report should contain whether the eligibility criteria, interventions and outcomes are identical (or very similar) to that of any trial that established efficacy of the reference treatment *(CONSORT statement extension, 2006*)

Note: The draft FDA guideline is not in effect yet and still open for changes (as per 18^th^ March 2010).

In this review, we described how published NI trials were conducted and reported, and whether the standard requirements from the guidelines were followed.

## Methods

### Search strategy and publication selection

**Figure 1:**
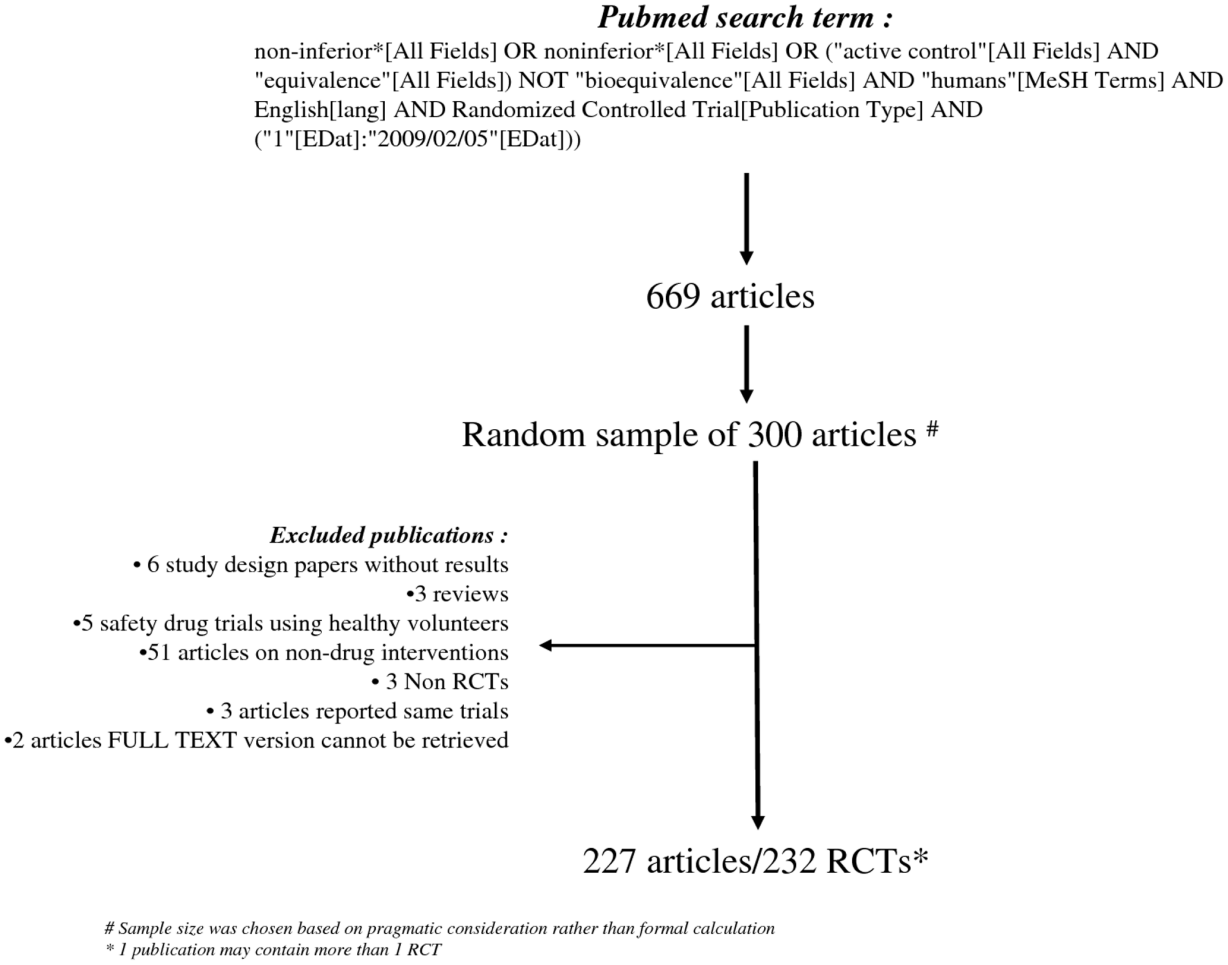
Flow diagram of trials' selection process.

We performed searches for NI trials in PubMed on 5 February 2009 and retrieved 669 articles as described in Figure 1. Subsequently, based on pragmatic consideration rather than formal sample size calculation, we used SPSS 16 to select a random sample of 300 articles. We subsequently excluded study design papers, reviews, trials using healthy volunteers, non-drug trials, non-RCTs, and articles of which the full-text version could not be retrieved. If one article reported multiple trials, we analyzed the trials separately. If multiple articles reported the result of one trial, we considered them as one subject, and included only the first publication.

### Data extraction

To extract relevant data, we created a standardized data extraction form, accompanied by an operational definition of each extracted variable. GW extracted all articles and MK extracted a randomly chosen 10% of the articles. GW and MK then compared the extraction results from the 10% articles. Disagreements occurred in seven articles and in three of 38 variables. The cause of the disagreements was the interpretation on vague information listed in the articles. We then decided that only a literal extraction was allowed, thus disallowing interpretation during extraction. For example for the degree of blinding, if only the description on how the investigator did the blinding but no clear terms e.g. double blind were written in the articles, we categorized it as ‘ambiguously stated’. We then updated the operational definition accordingly and GW rechecked the extraction results of those three variables in all the articles again and if necessary revised them.

For any missing information, if the articles referred to a registration database or previous paper for full description of the methods, information from these sources was retrieved.

### Characteristics of the trials

From each article, we extracted information on the journals' impact factor, type of drug, phase of the trial, trial's sponsor (independent investigator, pharmaceutical industry, or government), trial's design, primary endpoints, sample size, and the trial's conclusion of the new drug.

In addition, we extracted specific information whether the authors mentioned any additional benefit of the new drug and whether the additional benefit was addressed in the trial. For example, if the author mentioned that the additional benefit of the new drug was its better safety profile, we evaluated whether any formal safety profile comparison was included in the results section of the article.

We classified the journals based on their impact factor listed in the Journal Citation Reports® (JCR) 2008 edition. We arbitrarily chose a cut-off point of ten to classify the journal as high or low-impact.

We extracted the phase of the trial according to the statement in the publications or the referred clinical-trial's database e.g. clinicaltrials.gov. The classification was Phase I, II, III, and IV. Phase II and III might be divided into 2 parts, A and B. Phase IIA's primary aims are assessment and exploration of efficacy and pharmacodynamic aspects of the drug in patients with the target disease. In phase IIB, the main objectives are to confirm efficacy in a relatively large group of patients and determine optimal dose and dosing regimen to be implemented in phase III trials. In phase III trials, the main objectives are to confirm and to gather the additional information about the effectiveness and safety of the drug that are needed to evaluate the overall benefit-risk profile of the drug. Phase IIIA is conducted prior to application for marketing authorization, while phase IIIB is conducted after application. [14], [15]

We classified the type of primary endpoints as hard endpoints, intermediate endpoints and subjective endpoints. Hard endpoints were direct clinical events, such as mortality or stroke; intermediate endpoints were indirect outcome measurements that might not necessarily have a direct relationship with the clinical event such as laboratory data or biomarkers; and subjective endpoints are endpoints based on subjective perspectives of investigator or patient, such as quality-of-life questionnaires.

We extracted from the article specific characteristics of NI trials: degree of blinding, the method to determine the NI margin, the type of analysis, the use of a placebo arm to confirm assay sensitivity, and whether the authors discussed the constancy assumption. Furthermore, we extracted reasons for not including a placebo arm.

In terms of blinding, we extracted the literal term reported by the authors in the manuscript and classified the blinding into open-label, single, double, triple and “ambiguously stated” blinding.

Since there are guidelines on the NI margin for anti-infective drugs, we assessed within these trials whether their NI margin was consistent with them. Based on a guideline of the FDA (1992) and CPMP (1997), the recommended NI margin for anti-infective drugs is percentage difference of 10–20%.

We analyzed the quality of conducting NI trials by comparing the design and analysis characteristics of the trials reported in the high-impact vs. low-impact journals; and between the trials that were sponsored by industry and non-industry.

### Quality of reporting

To evaluate the quality of reporting, we compared the requirements from the extension of the CONSORT statement for NI and equivalence trials[10] between articles published before and after 2006 to evaluate the impact of the CONSORT statement extension on the reporting of NI trials. According to the extension of the CONSORT statement for NI trials, the method section should include additional information on how identical the inclusion and exclusion criteria, type of interventions and outcomes to previous efficacy trial of the active comparator were. The additional information should also include the NI margin and the method to determine it, sample size calculation, and whether a one-sided or two-sided confidence interval (CI) was used. The side of the CI is important in an NI trial as its inference of non-inferiority is based on the CI of the treatment difference between the test drug and its comparator. NI is concluded when the CI excludes and lays beyond the NI margin.[11] Furthermore, we compared the compliance to the CONSORT statement extension's requirements between trials reported in the high-impact and low-impact journals; and between the trials that were sponsored by industry and non-industry.

### Data analysis

Data were entered into a database using Epidata 3.1 (EpiData Association, Odense, Denmark; www.epidata.dk) and all statistical analyses were performed using SPSS 16 (SPSS Inc, USA; www.spss.com). The p-values for the differences were calculated using the Chi-square or Fischer's Exact test.

## Results

### Selection of the trials

The selection process of the NI trials is outlined in Figure 1. After filtering the articles based on the exclusion criteria, we included 227 articles in the analysis, which referred to 232 trials. One hundred eleven (47.8%) trials were published after 2006, the year in which the extension of the CONSORT statement on NI trials was published.

The missing data we retrieved from the registry were mostly data on the trial's phases and sponsorship. We only referred to the database as suggested by the author, so we believe the data in the register were reliable. We retrieved data of 34 trial's phases from clinicaltrial.gov; data of one trial's phase and data of one trial's sponsor from ISCRTN; data of one trial's sponsor from WHO international clinical trial registry; and data of one trial's phase from a sponsor clinical-trial registry website.

### The general characteristics of the trials

The general characteristics of the trials are described in Table 2. Most of the trials were published in low-impact journals (84.5%). Anti-infective drugs were the most studied drugs (22.9%).

**Table 2:** The general characteristics of trials.

	Number (%) unless stated otherwise
**Published in high-impact factor journals**	46 (19.8)
**Type of drug**	
Anti infective drugs	53 (22.9)
Cardiovascular and thrombolytic drugs	40 (17.2)
Drugs for endocrine disorders	26 (11.2)
Vaccines	24 (10.4)
Anti inflammatory and anti rheumatics drugs	17 (7.3)
Respiratory drugs	16 (6.9)
Neurological and psychiatric drugs	14 (6.0)
Anticancer drugs	11 (4.7)
Others	31 (13.4)
**Phase**	
Phase II	7 (3.0)
Phase III	69 (29.7)
Phase IV	12 (5.2)
Phase IIIB and IV	3 (1.3)
Not stated	141 (60.8)
**Sponsor**	
Independent investigator	39 (16.8)
Pharmaceutical industry	171 (73.7)
Government	6 (2.6)
Combination of any above	2 (0.9)
Not clear	14 (6.0)
**Design**	
Parallel	216 (93.1)
Cross-over	13 (5.6)
Factorial	2 (0.9)
Cluster-randomized	1 (0.4)
**Primary endpoints**	
Hard endpoints	97 (41.8)
Intermediate endpoints	102 (44.0)
Subjective endpoints	33 (14.2)
**Sample size** (median, interquartiles range)	
Number of planned subjects	388(242–673)
Number of subjects in ITT analysis divided by number of subjects planned	1.1 (1–1.2)
Number of subjects in PP analysis divided by number of subjects planned	1.0 (0.8–1.1)
**Conclusion**	
Non-inferiority was shown	209 (90.1)
Non-inferiority was not shown	17 (7.3)
Not clear	6 (2.6)

Almost one-third (29.7%) of the studies were phase III studies and the majority had pharmaceutical industry involvement in their trial process (73.7%).

Almost all studies had a parallel design (93.1%), and both hard and intermediate endpoints were often investigated. Variability between studies in the ratio of number of subjects in the analysis population versus the planned number of subjects was considerable. Most of the trials concluded that the new drug was shown to be non-inferior compared with its comparator (209 trials – 90.1%).

In 124 trials (53.4%), the authors mentioned additional advantages of the new drug. Most of the additional benefits mentioned and addressed were in terms of the safety profile of the drug, as shown in Table 4.

**Table 3:** Design and analysis characteristics.

	Number (%)
**Blinding method**	
Open-label	79 (34.0)
Single	18 (7.8)
Double	125 (53.9)
Triple	1 (0.4)
Ambiguously stated	9 (3.9)
**Method to determine NI margin**	
Based on investigator's assumption	51 (22.0)
Based on other publications or reviews	20 (8.7)
Based on guidelines	18 (7.7)
Calculated by the investigator based on previous trial's result	17 (7.3)
Not clear	126 (54.3)
**Type of statistical analysis**	
Both ITT and PP	97 (41.8)
Only ITT	81 (34.9)
Only PP	46 (19.8)
Not clear	8 (3.5)
**Including placebo-arm to confirm assay sensitivity**	14 (6.0)
**Discuss constancy assumption**	9 (3.9)

### The quality of conducting NI trials

**Table 4:** Additional benefit of the new drug mentioned in the publication.

% *	N,	Addressed¶ (% from N)
Better safety profile	45 (36.3)	43 (95.6)
Better method of administration	19 (15.4)	6 (31.6)
Better safety profile and method of administration	12 (9.7)	10 (83.3) †
Better method of administration and induce higher patient's compliance rate	12 (9.7)	5 (41.7) ‡
Better safety profile and induce higher patient's compliance rate	7 (5.6)	5 (71.4) §
Induce higher patient's compliance rate	6 (4.8)	3 (50.0)
Better method of administration and low cost	5 (4.0)	2 (40.0) ¥
Others than above	18 (14.5)	10 (55.6)

*Note:*

*Percentage is based on 124 trials that mentioned any additional benefit of the new drugs irrespective of whether or not data were shown to support the claim.

¶ The authors show any analysis or argument of the additional benefit.

† Three trials addressed both the safety profile and the method of administration, six trials only addressed the safety profile, and one trial only addressed the method of administration.

‡ One trial addressed both the method of administration and patient's compliance rate, and four trials addressed only the patient's compliance rate.

§ Four trials addressed both safety profile and patient's compliance and one trial only addressed the safety profile.

¥ One trial addressed both better method of administration and cost and one trial only addressed cost.

**Table 5:** Stratification of the articles according to their journal impact factors.

Trials' design and analysis issues	High-impact (N = 46)	Low-impact (N = 180)	p value
	N (%)		
**Blinding method**			0.11
Open-label	20 (43.5)	56 (31.1)	
Single	5 (10.9)	12 (6.7)	
Double	18 (39.1)	105 (58.3)	
Triple	0 (0)	1 (0.6)	
Ambiguously stated	3 (6.5)	6 (3.3)	
**Method to determine NI margin**			0.34
Based on investigator's assumption	16 (34.8)	35 (19.4)	
Based on other publications or reviews	5 (10.9)	14 (7.8)	
Based on guidelines	2 (4.3)	16 (8.9)	
Calculated by the investigator based on previous trial's result	3 (6.5)	14 (7.8)	
Not clear	20 (43.5)	101(56.1)	
**Type of statistical analysis**			0.01
Both ITT and PP	14 (30.4)	80 (44.4)	
Only ITT	25 (54.3)	56 (31.1)	
Only PP	6 (13.2)	39 (21.7)	
Not clear	1 (2.1)	5 (2.8)	
**Including placebo-arm to confirm assay sensitivity**	2 (4.3)	12 (6.7)	0.74
**Discuss constancy assumption**	1 (4.3)	7 (8.9)	0.65

The design and analysis characteristics of the trials are described in Table 3, while stratification according to journal impact factors is shown in Table 5. Six journals did not have their impact factor listed in the JCR 2008 edition and were not included in the analysis. We found no significant difference in terms of trials' characteristics between trials that were sponsored by pharmaceutical industry or not (data not shown).

More than half of the trials were stated as double blinded, while a substantial number (79, 34.0%) was open label. We found no difference in terms of blinding method between trials that were published in high-impact or low-impact journals.

We observed that 227 (97.8%) trials reported their NI margin in the articles. Nevertheless, only 106 (45.7%) trials reported the method by which the NI margin was determined. In 51 (22%) trials, the margin was determined merely based on investigator's assumption. In 20 (8.7%) trials, the NI margins were obtained from other publications or reviews. In 18 (7.7%) trials, the NI margins were obtained from guidelines and in 17 (7.3%) trials the NI margins were calculated by the investigators based on data from previous trials. Among the last, 15 of them used a preserved fraction of 50% or greater. We also found in 95 (40.9%) trials, the authors mentioned that the NI margin was a clinical acceptable margin. Among them three trials mentioned that the decision to use the margin was validated by a panel of clinical experts. We found no difference in terms of method to determine the NI margin between trials that were published in high-impact or low-impact factor journals.

Within 53 anti-infective trials, most of the trials (42, 77.8% of all anti-infective trials) used an NI margin of percentage difference between 10 to 20%. Only four trials used a NI margin less than 10% or more than 20%. In the rest of seven trials, six trials did not use percentage difference as an NI margin, and in one trial, the NI margin was not clear.

In terms of statistical analysis, most of the trials (127, 54.7%) used either ITT or PP, while 97 (41.8%) trials used both ITT and PP analysis. We found among the trials that used both ITT and PP analysis, 94 of them concluded that the new drug was non-inferior to its comparator. In 53 trials of the latest, the conclusions were deducted from similar results of both ITT and PP analysis. In the rest of the trials: 22 trials concluded non-inferiority based on the results of their PP analysis; 18 trials were based only on the results of their ITT analysis; while in three trials, it was not clear on which analysis their conclusion was based. We found a significant difference in terms of type of statistical analysis between the trials published in high-impact and low-impact factor journals. Trials published in the high-impact journals mostly used only ITT analysis (54.3% of 46 trials), while in the low-impact journals, both analysis methods were most frequently used (44.4% of 180 trials).

In our review, we observed that 210 trials (90.5%) did not include a placebo arm to confirm assay sensitivity. Only 19 trials mentioned the reason why a placebo arm was not included in trials, and almost half of them were due to ethical reasons. We observed that the inclusion of a placebo is quite common (28.6%) in trials with neurology/psychiatric drugs. This is probably because in this type of drugs, the constancy assumption will often not hold, as the placebo effect in previous placebo-controlled trials is difficult to rule out. In addition, we found no difference in terms of using a placebo arm to confirm the assay sensitivity between trials that were published in high-impact or low-impact factor journals.

Additionally, we observed only nine (3.9%) authors discussed the constancy assumption and there was no difference in this respect between trials that were published in high-impact or low-impact journals.

### Compliance in reporting NI trials

Only 3.0% of the trials reported the similarity of the inclusion and exclusion criteria with previous trials studying the effect of the active comparator, 5.6% of the trials reported the similarity of the type of intervention with previous trials, and 3.4% of the trials reported the similarity of the outcomes. Seventy-seven (33.2%) trials did not report whether they were going to present the data using one-sided or two-sided CI in the methods section as required by the CONSORT statement. Furthermore, we found that the papers in low-impact journals reported the side of the CI more frequently than those in the high-impact factor journals, and the difference was significant (Table 6).

**Table 6:** Comparison of reporting of essential information in NI trials.

Reported in the method section of a NI article	*Between High-impact journals and low-impact journals*			*Before and after CONSORT statement in 2006*		
	Percentage of trials		p-value	Percentage of trials		p-value
	High-impact (N = 46)	Low-impact (N = 180)		Before and until 2006 (n = 121)	After 2006 (n = 111)	
Eligibility similarity	2.2%	2.8%	1.00	2.5%	3.6%	1.00
Type of Intervention similarity	10.9%	3.9%	0.07	5.8%	5.4%	0.90
Outcomes similarity	4.3%	2.8%	0.63	2.5%	4.5%	1.00
NI margin	97.8%	98.3%	1.00	97.5%	98.2%	1.00
Method to determine NI margin	56.5%	43.9%	0.12	50.4%	40.5%	0.15
Side of CI	50.0%	71.7%	<0.01	64.5%	69.4%	0.26

The compliance in reporting the items required by the extension of the CONSORT statement before and after 2006 is described in Table 6. We did not observe improvement of reporting after the release of the CONSORT statement extension for NI trials. The method of determination of the NI margin was even reported less frequently in trials published after 2006 than in trials published before and in 2006.

## Discussion

In this review, we found five main issues in the design, analysis and reporting of NI trials. First, many of the trials were open label trials. Second, reporting the method to determine the NI margin was infrequent and limited. Third, most of the trials analyzed their data with one statistical analysis method; ITT or PP. Fourth, we observed that only few trials included a placebo-arm to confirm assay sensitivity and that only few trials discussed the constancy assumption. Lastly, we did not observe any difference in terms of reporting in NI trials published before or after the release of the extension of the CONSORT statement for NI trials in 2006.

In our review, about a third of the trials were open label trials. This surprising finding was not consistent with the guidelines[8], [11] that suggest to use blinding whenever possible to minimize the risk of bias. This leads to discussion on the importance of blinding in an NI trial. Snappin believes that blinding only gives minor protection in NI trials, since a blinded investigator with a preliminary belief in non-inferiority of the test drug can bias the result by assigning similar ratings to the treatment responses of all patients. [16] There is no doubt, however, that blinding does offer protection against information bias. In addition, there will usually be endpoints (e.g. safety) for which differences are expected and for which blinding will ensure stronger evidence. We therefore conclude that blinding is still important in NI trials to avoid bias. If blinding is not possible, subjective endpoints need to be avoided and more stringent monitoring should be conducted.

The method to determine the NI margin was not reported in more than half of the trials. This finding is consistent with previous reviews in 2005 to 2006, where the methods were presented in 46% or less of the trials.[17], [18], [19] Apparently, the extension of the CONSORT statement in 2006 has not brought any significant impact yet. Furthermore, the statement has suggested that the NI margin should be preferably justified on clinical grounds and its relation to the effect of the reference treatment relative to placebo in any previous trials should be noted.[10] We found that most of the authors included a statement that the NI margin was a clinically acceptable difference, but only three trials mentioned that the margin was validated by a panel of clinical experts. This finding was consistent with other reviews[17], [18], [19], where many trials claimed that their margin was clinically relevant without any clear details how the clinically acceptable NI margin was chosen. Putting merely a statement that the margin was determined based on clinically acceptable difference is not sufficient for any subsequent trial replications. Thus, more details are needed in the description on how the NI margin was determined. Furthermore, a detailed description on how the margin was determined can help the reader to decide whether the NI margin and the rationale for the margin's choice influenced the validity of the results.

We observed in anti-infective drug trials, that most of them used a constant difference of 10–20% in treatment difference as their NI margin. Regulators recommend an NI margin of 10% for vaccines and anti-bacterials. [20], [21] This margin of 10% is acceptable as long as the primary outcome of interest has a high incidence rate. The implication of using a 10% constant margin in vaccines and anti-infective drugs should be further explored and any improvement on the guidelines to determine NI margin should cover this issue.

We observed that most of the trials reported the result only from ITT analysis or PP analysis. Our results were consistent with a previous review that observed that more NI trials used ITT rather than PP.[18] We also observed that ITT analysis was more reported in high-impact journals. The CPMP guidelines and the new draft FDA guidelines for NI trials already stated that both analyses have equal importance in NI trials. For superiority trials, ITT analysis is the preferred analysis as it adheres to randomization [11] and might best reflect clinical practice. PP analysis might violate randomization and not reflect clinical practice very well. Several reviews with RCT simulation showed that both ITT and PP could be problematic in NI trials, especially if the trial had large number of non-compliance.[22], [23], [24] In addition, in our data, we did not observe any evidence that ITT will lead to more NI conclusions than PP. We conclude that both analyses are equally important, as each approach brings a different interpretation for the drug in daily practice.

We observed that only a small number of trials included placebo arms to support assay sensitivity. Although our data did not provide sufficient evidence whether the use of placebo was appropriate or not in the trials, we believe that the use of a placebo arm was probably not ethically feasible in most studies. Nonetheless, the non-inferiority result of the drugs in NI trials might bear two meanings: both drugs are equally effective, or both drugs are equally ineffective against placebo. In this sense, a placebo arm in an NI trial will enable evaluation whether both drugs in the trial are effective, if the trial shows non-inferiority. Alternatively, if the use of a placebo arm is not possible, the trial should choose a margin that assures that the estimated effect of the new drug is likely to be superior to placebo, under the constancy assumption for the active comparator. The readers, not only the investigators, also need to be aware of this issue of assay sensitivity in interpreting the result of NI trials. They need to consider the type of endpoints; the number of patients in the final analysis; reasons of patient's dropouts; the similarity of the trial with the previous trial(s) that established the efficacy profile of the comparator; and the constancy assumption of the data used as reference for the NI margin. Based on our review, two of the latter were only being reported in a small numbers of the articles.

Less than five percent of the trials in our review mentioned whether the trials were designed similar to relevant past trial(s). Thus, it was difficult to assess whether the historical data that were used for determining the NI margin were reliable. Since the validity of the NI margin is related to the interpretation of the NI trials, clear reporting of the method of NI margin determination and the constancy assumption is essential for every NI trial publication. It is impossible to check the validity of the constancy assumption without a parallel placebo arm. However, at minimum, it is possible to check whether the current NI trial was similar to previous trial(s) that estimated the efficacy of the active comparator. [25]

We found no difference between reporting before and after the release of the extension of the CONSORT statement on NI trials. Furthermore, in general, there is no difference in adherence to the CONSORT statement between the high-impact and the low-impact journals. The overall low adherence to the statement might be due to unfamiliarity of the authors, referees, and editors of all of the journals with the statement extension. Researchers and editors of journals should be more aware of this extension and should comply with its recommendations. We realized that it might be too early to see full adherence of the CONSORT statement extension after 3 years, but due to the reputation of the CONSORT statement itself, we considered it reasonable to expect a certain degree of improvement.

Our review has some limitations. First, we excluded several trials since we only used a random sample of all NI trials that we identified. However, as this was a random sample, this will not have influences our results. Second, we only used PubMed to identify NI trials; therefore, we might have missed some trials. However, we assume that NI trials retrieved from PubMed do not have different methodological characteristics than NI trials in other databases, so we do not think that this influenced our results. Third, since the terms that we used to search for non-inferiority trials were not standard MESH terms and our search for those terms was limited to the abstract of the articles, our search might not have captured all NI drug trials available in PubMed. Also for this selection, we expect that the NI trials that we found are not different from the NI trials that we did not capture with our search. A strength of our study is that we did not only focus on the NI margin, as previous reviews[17], [18], [19] did, but also evaluated other methodological aspects of NI trials. In addition, we evaluated the quality of reporting using the current guidelines from the CONSORT statement.

In conclusion, the conduct and reporting of NI trials can be further improved. Particularly, in terms of maximizing the use of blinding, the use of both ITT and PP analysis, reporting the similarity with the previous comparator's trials to guarantee a valid constancy assumption and reporting the method to determine NI margin.

## References

[1] Hung HMJ, Wang S-J, O'Neill R (2005). A Regulatory Perspective on Choice of Margin and Statistical Inference Issue in Non-inferiority Trials.. Biometrical Journal.

[2] D'Agostino R, Massaro JM, Sullivan LM (2003). Non-inferiority trials: design concepts and issues - the encounters of academic consultants in statistics.. Statistics in Medicine.

[3] (2009). Regulatory watch: Non-inferiority-trial discussions impact new drug applications.. Nat Rev Drug Discov.

[4] Garattini S, Bertele V (2007). Non-inferiority trials are unethical because they disregard patients' interests.. The Lancet.

[5] Jones B, Jarvis P, Lewis JA, Ebbutt AF (1996). Trials to assess equivalence: the importance of rigorous methods.. BMJ.

[6] Senn S (2007). Active control equivalence studies. Statistical Issues in Drug Development. 2nd ed. Glasgow: John Wiley & Sons, Ltd..

[7] Kay R (2007). Equivalence and non-inferiority.. Statistical thinking for non-statisticians in drug regulation.

[8] ICH (2001). E10: Choice of Control Group and Related Issues in Clinical Trials.

[9] Koch A, RÃhmel J (2004). Hypothesis Testing in the “Gold Standard” Design for Proving the Efficacy of an Experimental Treatment Relative to Placebo and a Reference..

[10] Piaggio G, Elbourne DR, Altman DG, Pocock SJ, Evans SJW (2006). Reporting of Noninferiority and Equivalence Randomized Trials: An Extension of the CONSORT Statement.. JAMA.

[11] ICH (1998). Statistical principles for clinical trials - E9.. In: Use ICOHOTRFOPFH, editor: ICH Expert Working Group.

[12] CHMP (2005). Guideline on the Choice of the Non-Inferiority Margin..

[13] CDERCBER Draft Guidance: Guidance for Industry Non-Inferiority Clinical TrialsIn: Services HaH, ed: Food and Drug Administration; 2010:66.. http://www.fda.gov/downloads/Drugs/GuidanceComplianceRegulatoryInformation/Guidances/UCM202140.pdf.

[14] CDER (2003). Inside Clinical Trials: Testing Medical Products in People FDA Consumer Magazine.

[15] ICH (1997). General Considerations For Clinical Trials E8..

[16] Snapinn SM (2000). Commentary: Noninferiority Trials.. Curr Control Trials Cardiovasc Med.

[17] Lange S, Freitag G (2005). Special Invited Papers Section: Therapeutic Equivalence - Clinical Issues and Statistical Methodology in Noninferiority Trials.. Biometrical Journal.

[18] Le Henanff A, Giraudeau B, Baron G, Ravaud P (2006). Quality of Reporting of Noninferiority and Equivalence Randomized Trials.. JAMA.

[19] McCaffrey N, Merlin T, Hille J (2006). Lack of clinical rationale provided for non-inferiority margins..

[20] CPMP (2003). Note for guidance on evaluation of medicinal products indicated for treatment of bacterial infections..

[21] CPMP (1999). Note for guidance on clinical evaluation of new vaccines..

[22] Brittain E, Lin D (2005). A comparison of intent-to-treat and per-protocol results in antibiotic non-inferiority trials.. Statistics in Medicine.

[23] Sanchez MM, Xun C (2006). Choosing the analysis population in non-inferiority studies: per protocol or intent-to-treat.. Statistics in Medicine.

[24] Sheng D, Kim MY (2006). The effects of non-compliance on intent-to-treat analysis of equivalence trials.. Statistics in Medicine.

[25] Fleming TR (2008). Current issues in non-inferiority trials.. Statistics in Medicine.

